# It’s hard to play ball: A qualitative study of knowledge exchange and silo effects in public health

**DOI:** 10.1186/s12913-017-2770-6

**Published:** 2018-01-02

**Authors:** Rebecca Johnson, Amy Grove, Aileen Clarke

**Affiliations:** 1 Collaboration for Leadership in Applied Health Research and Care West Midlands, Warwick Medical School, University of Warwick, Coventry, CV4 7AL UK; 2 Division of Health Sciences, Warwick Medical School, University of Warwick, Coventry, CV4 7AL UK

**Keywords:** Knowledge exchange, Public health partnership, Silos, Qualitative, Thematic analysis

## Abstract

**Background:**

Partnerships in public health form an important component of commissioning and implementing services, in England and internationally. In this research, we examine the views of staff involved in a City-wide health improvement programme which ran from 2009 to 2013 in England. We examine the practicalities of partnership work in community settings, and we describe some of barriers faced when implementing a large, multi-organisation health improvement programme.

**Methods:**

Qualitative, semi-structured interviews were performed. Purposive sampling was used to identify potential participants in the programme: programme board of directors, programme and project managers and intervention managers. Interviews were conducted one-to-one. We conducted a thematic analysis using the ‘one sheet of paper’ technique. This involved analysing data deductively, moving from initial to axial coding, developing categories and then identifying emerging themes.

**Results:**

Fifteen interviews were completed. Three themes were identified. The first theme reflects how poor communication approaches hindered the ability of partnerships to deliver their aims and objectives in a range of ways and for a range of reasons. Our second theme reflects how a lack of appropriate knowledge exchange hindered decision-making, affected trust and contributed to protectionist approaches to working. This lack of shared, and communicated, understanding of what type of knowledge is most appropriate and in which circumstance made meaningful knowledge exchange challenging for decision-making and partnership-working in the City-wide health improvement programme. Theme three demonstrates how perceptions about silos in partnership-working could be problematic, but silos themselves were at times beneficial to partnerships. This revealed a mismatch between rhetoric and a realistic understanding of what components of the programme were functional and which were more hindrance than help.

**Discussion:**

There were high expectations placed on the concept of what partnership work was, or how it should be done. We found our themes to be interdependent, and reflective of the ‘dynamic fluid process’ discussed within the knowledge mobilisation literature. We contend that reframing normal and embedded processes of silos and silo-working already in use might ease resistance to some knowledge exchange processes and contribute to better long-term functioning of public health partnerships.

## Introduction

Knowledge exchange has been defined as “collaborative problem solving between researchers and decision makers that happens through linkage and exchange” [1] and also as “a dynamic and fluid process which incorporates distinct forms of knowledge from multiple sources” [2]. In public health it can be conceptualised in a range of ways [3] and with a range of dimensions [4].

Knowledge exchange is predicated on the idea that individuals or groups come together as communities to exchange ideas, evidence and expertise. Therefore, any activity which reduces communication, hinders access or diminishes the clarity of information hinders knowledge exchange and leads to poor sharing across communities in practice.

Partnership in Public Health is a necessary mechanism to implement most types of health intervention. Partnership can be defined as a relationship between individuals or groups that is characterised by mutually agreed collaboration, responsibility and action to meet the objectives of a particular aim. In England, changes to the structure of service delivery, such as the transition of Public Health teams from the NHS into local authorities in 2013, have created a wider range of partnerships, including collaboration between private, charitable and public sectors. While these partnerships offer many opportunities for knowledge exchange, they also carry the potential for extending the challenges public health departments already face, thus further complicating an already complex network of relationships. For example, there is evidence that partnerships in public health create language barriers, silo working, and the prioritisation of different outcomes [5]. The practicalities of partnership in public health, and what to do about them, are neither clear nor simple.

In this article, we examine the views of staff involved in a City Health Improvement Programme (hereafter named CHIP) and describe key aspects of knowledge exchange in their public health partnerships which facilitated and hindered the developing relationships.

## Background

In 2010, the NHS white paper ‘Equity and Excellence: Liberating the NHS’ [6] marked a sea-change for the NHS and Public Health in England by outlining changes to the way services were to be delivered in England. This included the allocation of funds designated for population wide health improvement programmes.

Reflecting the prescience of those working in public health in City Primary Care Trust (PCT) and City Council, these two organisations developed the City Health Improvement Programme (CHIP), which ran from 2009 to 2013. It comprised nine projects which aimed to improve health outcomes for residents of the City, while reducing health inequalities. In CHIP, Local Authority and NHS partners worked together on a series of health interventions delivered in the community, pre-empting yet closely reflecting the Public Health transition to local authorities.

### Study context, health in the ‘City’

Prior to and during the implementation of CHIP, the health of people who live in the City was worse than that for England overall. There were higher rates of smoking during pregnancy, more early deaths from cancer, a significantly greater proportion of obese adults and obese children, and significantly lower rates of physical activity among adults. There was therefore a need to increase healthy lifestyle behaviours in City. As one part of a multi-method evaluation of CHIP. The name of this city has been anonymised to maintain confidentiality.

## Methods

### Design

In-depth qualitative case study. We used face-to-face, semi-structured interviews to collect data.

### Participants and setting

A ‘CHIP stakeholder’ was defined as a person involved in the organisation, management or implementation of CHIP interventions. These included public health directorate members, project managers and intervention managers, from which the purposive sampling frame was identified. These potential participants included people affiliated with both the NHS and local authority (LA). Equal numbers of people were invited to be interviewed: CHIP directorate (6 invited), project managers (6 invited) and intervention managers (5 invited).

### Interviews

Interviews took place between March 2012 and May 2012 and were digitally recorded. Our interview guide focused on three topic areas: implementation and evaluation, public health improvement practice, and mental wellbeing outcomes. The guide was divided into two parts. The first part included questions adapted from each domain of the RE-AIM framework for improving public health evaluation: Reach, Effectiveness, Adoption, Implementation, Maintenance [7]. The second part included unstructured questions about the interviewees’ overall experiences of partnership working during CHIP. This article focuses on interviewees’ overall experiences of partnership working.

### Validation and coding

Issues common to the reliability and validity of qualitative data were addressed [8]. The interview guide was revised after the first three interviews to ensure it flowed coherently to ensure maximum suitability for interviewees. Each interviewee was given an ID number to maintain confidentiality. Interviews were transcribed verbatim. One interview from each group (*n* = 3) was open coded line-by-line. From these three open-coded interviews, 75 codes were generated. We assessed the similarities and differences between codes and expanded or combined them where appropriate. Sixty codes were finalised within 12 categories, which we used to develop our analysis.

### Data analysis

Data were analysed using the ‘One Sheet of Paper’ technique or ‘OSOP’ developed by Ziebland and McPherson [9]. Data were analysed deductively, moving from initial to axial coding, developing categories and then identifying emerging themes For each category, issues were identified from a coding summary, and these issues were transformed into three themes. Each theme was developed through an iterative process of refining and expanding emerging concepts and issues. The similarities and differences, discriminant cases, gaps in views and points of views between and within categories, groups of interviewees and each individual interviewee were considered. Quotations from the original interviews were accessed to illustrate and cross-check the consistency of themes. A final cross-checking of OSOP issues with the original interview transcription was undertaken to ensure we did not deviate from any theme, or the theme had not ‘drifted’ from the raw content [8].

## Results

Of 17 requests for interview, 15 staff were interviewed and two declined. There was a balanced number of participants from the NHS (*n* = 7) and local authority (*n* = 8).

Participants described the aim of CHIP; ‘*to put health improvement on the public health agenda*’, ‘*to create opportunities for health improvement innovation*’, and to ‘*bridge the gap between local authority/city council and the NHS*’. Staff described feeling that CHIP was ‘*exciting*’ (A15, B3, B6) and that it was a ‘*fantastic opportunity’* (B3, B5, B1). These feelings of excitement and optimism were coupled with the notion that CHIP was also ‘*a nightmare*’ and ‘*frustrating*’ (P4,P5, A15, A11) and people felt ‘*rushed*’ into the process (A11,A15,B3,P6) at the beginning.

In our findings, we have characterised definitions of partnership-working in CHIP, and identified three interdependent themes from our analysis. The first theme reflects how poor communication approaches in CHIP hindered the ability of partnerships to deliver their aims and objectives. Our second theme reflects how a lack of appropriate knowledge exchange hindered decision-making. Finally, theme three demonstrates how perceptions about silos in partnership-working could be problematic, but silos themselves were beneficial to partnerships. Together these themes demonstrate a network of problematic approaches to partnership. Poor communication led to a lack of clarity about which types of knowledge should be drawn on, to make decisions. This lack of appropriate knowledge exchange eroded trust, wasted time, and reinforced negative perceptions of silo-working.

### Defining good partnership-working

Good partnership-working in CHIP was achieved through positive working relationships within and between teams. It was characterised by enthusiasm, clarity of communication, willingness to compromise and a sense of ‘team spirit’. The sense of a good partnership was established over time, and built trust through open discourse and the mutual beneficence by members regarding project work. As represented below:

*“I love the team, erm, that’s been built. ‘Cause I think we work really well together. And they’ve got such good knowledge of the people who work in the area. And I think that makes a massive difference. So we’re kind of like, we had this storming and norming phase where we were getting people trained up and now, kinda like, we’re running with it. And that’s such a good feeling.”* (A11)

Partnership-working described as ‘good’ supported intervention delivery and helped to establish and extend knowledge and skills among staff. Developing practical ways to work strategically and sustainably was seen by interviewees as important, and related to good partnership-working. Contrastingly, there were three main barriers to good partnership-work and they are discussed below.

### Theme 1. Poor communication approaches

Poor approaches to communication in partnership-working were characterised by linear, rigid and top-down communication styles. These poor approaches involved interpersonal politics, the wielding or pursuit of power and status, vague project planning objectives, culturally different organisational practices and the use of unclear terminology. The poor communication we found did not necessarily prevent work from happening, but exacerbated the use of time and energy spent ‘fire-fighting’ problems that resulted from poor communication and reduced the capacity of staff to perform anything other than their necessary duties. This left little time for the positive communication approaches, including the sharing of technical knowledge and experiences, clarifying terminology, organisational processes or common practices of distinct groups. Poor communication also left little space or time for a relaxed and engaged atmosphere that lent itself to serendipitous bonding and knowledge brokering between individuals and teams. Participants described an atmosphere of ‘*brinkmanship*’ (P4) and protectionist approaches to partnership:

*“No, they don’t even want to work with each other, it feels like. There’s so much politics in that work stream... ‘Cause everyone seems to want to be the chief. In that area....And no one’s really playing ball, it feels like.” (A11)*

*“...To a degree there’s an element of people wanting to misinterpret because they wanted an excuse to hold on to the clients...And I think that is a barrier.” (P8)*

Political aspects of working in partnership were not discussed positively by any participants, and were described as explicit barriers to partnership, a sign of lost potential, and a lack of professionalism. Top-down communication approaches between management and delivery staff exemplified poor communication approaches when one-way communication between management levels occurred, or feedback from ‘bottom to top’ went unspoken or unanswered. A strong tradition of measuring outputs rather than outcomes in both CHIP organisations created new and unexpected challenges in managing the change from the medical model perspective to a more context-relevant, holistic logic of care that is seen in the Local Authority.

The presence and absence of shared meaning around key concepts was a source of confusion and sometimes conflict among individuals and wider organisations throughout CHIP. Multiple intangible concepts proved challenging in practice, such as; commissioning, procurement, health outcomes (as opposed to outputs) and evaluation. These concepts provoked a challenge for two main reasons. Firstly, the culturally normative terms or concepts differed between organisations. As demonstrated in the quote below;

*“Although it was a kind of NHS initiative at the start it was run from within the city council, who I think had very different processes, all their procurement rules and things like that were different. That wasn’t really thought in enough detail at the beginning...I think it created problems and delays throughout the process.”* (B5)

Secondly, these concepts were relatively new for some staff. They had been introduced with implicit assumptions of the levels of staff knowledge, capability and time to make sense and establish a shared meaning.

*“And it was just horrendous. And each PID [Project Initiation Document], you didn’t actually know what a PID was and how long a PID was meant to be*. W*e were expected to have written our PIDs, allocated the money, written the service level agreements, while also being told all these project management things that we had to do.” (A15)*

Communication approaches in CHIP partnership-working caused delays and confusion through a lack of clarity and a lack of shared understanding. This lack of shared understanding, meaning and language between project staff, managers, and directors hindered knowledge exchange for the partnership.

### Theme 2. Lack of appropriate knowledge exchange

Our second theme focuses on the knowledge that was exchanged within the partnership. Knowledge exchange typically refers to the exchange of formal research evidence sharing between research producers and research users. Here we use this term more flexibly, to include any type of knowledge exchanged between organisations and teams operating within the partnership. They exchange knowledge with the purpose of making an informed decision. We found the term ‘evidence’ used as an umbrella term for knowledge of all kinds. The exchanging of different types of knowledge, at key times points in the programme, was important to how staff discussed the operational success of the CHIP partnership in our study. We identified three distinct perceptions of knowledge products being exchanged within the partnership: research evidence, practical experience and know how.

Participants discussed knowledge, experience and evidence strategically, as part of a plan for sustainability of their projects. For some, evidence seemed to matter because it enabled staff to make explicit what they already implicitly knew from their own experience. They knew that their projects worked, they just needed to evidence this by demonstrating it tangibly to others. In this way evidence mattered only for proving the worth of the intervention, not whether the intervention was evidence-based in the clinical science perspective, or whether it demonstrated effectiveness after formal evaluation.

This notion of proof revealed two interesting findings. Firstly, that a tension existed between staff delivering interventions at the coal face, and witnessing its success, but struggling to come to terms with a lack of tangible proof that this was the case. Secondly, it revealed a lack of understanding of the different forms of knowledge and more formal uses and expectations behind the term evidence. See the quotes below to demonstrate this point;

*“…it’s one of those things that you feel and you know, but is that going to be received? The people want the, academic and the statistical proof that actually it is in place and it has [worked].” (A12)*

*Erm, I don’t know the research on it...But from my experience, of the cases that I worked with I have seen people use it to their benefit. (A13)*

*“And just because at that time when they went on your programme there wasn’t this, erm, light bulb moment, it doesn’t mean it didn’t make a difference to their lives....But it goes back to the question of well okay then, prove it. So how you prove it, that’s the bit I don’t know.” (A11)*

Evidence was a powerful element in the meaning associated to CHIP. It appeared to influence confidence and increase worries about project sustainability, affect ‘good’ and ‘poor’ commissioning decisions, influence how staff approached their interventions, and the personal stakes invested in those interventions. In this sense, the meaning of evidence in CHIP was centered on the strategic utility of evidence, i.e., its ability to justify the continued existence of a given project. While the rhetoric and intention for evidence-based interventions was there, in essence this was less well observed in practice;

*“I mean… everything we do in the health service isn’t evidence based… whilst we have …these high principles, erm, the reality is that most of the time we do stuff ‘cause we think it’s a good idea.” (B6)*

The second type of knowledge we discovered being used in CHIP was practical experience. There was a sense of dissatisfaction with how managers undervalued intervention deliverer’s practical knowledge;

*“So they’ll tell you what to do. But there doesn’t seem to be any leeway to say well actually we’re on the ground and I don’t think that’s gonna work.” (A11).*

This lack of two-way exchange illustrates a misunderstanding of when and how best to utilise different types of knowledge in partnerships. The expert knowledge of process and practice-based context held by project deliverers was ignored and caused a lack of trust between project managerial teams and those responsible for delivering it. It was this lack of shared understanding of what type of knowledge is most appropriate and in which circumstance that made knowledge exchange problematic for decision-making and partnership-working in CHIP.

Another form of knowledge we identified was know-how. In our study this represents the practical and technical skills needed for managing, delivering and evaluating interventions in the real world practice of public health. There was ‘*huge variation*’ (P5) in the technical and professional skill available in CHIP. Evaluation was a difficult concept for some staff to grasp, and it was often portrayed as requisite but undesirable. Participants felt that evaluations marginalised the ability of project staff to their job, while some managers introduced evaluations when staff felt they were already at capacity;

*“And it was like another thing that we were being asked to do and it was it wasn’t very well received by staff...” (A13).*

In terms of knowledge exchange, evaluations were an opportunity to learn and revise a project approach to improve health outcomes. But assumptions about what evaluation should mean, and in practice what evaluation actually meant, coupled with the know-how to complete them, fostered a challenging atmosphere for knowledge management in the partnership;

*“And perhaps that irascibility is kind of unfair because I’m assuming that people are at a slightly different level to what I think they should be at. And again it’s that cultural mismatch in terms of knowing what an evaluation should be about … and almost, err, finding it difficult to entertain the notion that people don’t understand evaluation*.” (B1)

*“...But, err, we did, err, [external organisation] evaluated our programme and the aim of that was to get some tools that these kinds of programmes could use, but the stuff was really academic...The people we work with, literacy levels are really low, they’re not gonna understand some of the stuff, so it was completely useless.” (A11)*

We found that expectations for CHIP partnership-working were predicated on a healthy exchange of different types of knowledge in theory, such as wider CHIP objectives encouraging partnership, sharing learning, creating innovative projects, and being evidence-based. CHIP practice however, reflected knowledge exchanges that were often decoupled from these objectives. Bad timing, poor management, insufficient capacity and technical understanding of staff responsibilities all hindered the partnership. There seemed to be a misunderstanding of when to make use of certain kinds of knowledge: evidence, experience, and know-how.

Each type of knowledge was not always utilised at the most appropriate time for a given project, or communicated well between project delivery groups or between staff and managers. These gaps acted as boundaries in knowledge exchange between groups and therefore hierarchies stagnated the growth of partnership-working in CHIP. They also promoted silo-working approaches that were exacerbated by the poor communication approaches presented in theme one.

### Theme 3. Silos in partnership-working

In this research the term silo refers to projects or teams that worked in isolation, who did not appear to engage with other projects or the programme as a whole. One interviewee illustrates the possible origin for early entrenchment of silo-working, stating how the early bureaucratic style of higher management was seen as “unrealistic” and that it “alienated” and “undermined” what could be seen as a more naturalistic approach to project work: *“we were thinking what is it we’re trying to do? What are we trying to achieve?”* whist being told to develop a project initiation document without having “done the scope” which “got things off on a bad footing” (B4).

Working in silos was discussed as a negative aspect of partnership working which appeared to hinder the building of good partnerships. The discussion of silos in our interviews brought forth dialogue regarding what exactly partnership-working was or how staff were expected to do it. This reflected an assumption that good partnership should have come more effortlessly than it did, as described below;

*“The projects are operating in silos, for the most part. And we have tried to cross link them but it hasn’t been as effective as we’d like. And I can understand why. It’s not easy.”* (B1)

*“And I don’t think it was ever really developed. I think it was only at a kind of fairly late stage where people started trying to push some cross working.” (P8)*

*“...And it’s that side of things, the power and control I think is just so important to them rather than team working and partnership-working.” “…they were very reluctant to change their direction and, I think, they saw a loss of power.” (P5)*

Silos reflected a lack of knowledge about other practices, but also a desire and awareness to know what other CHIP practices were going on;

“*Yeah. I don’t know. I don’t know. Yeah, I mean, there are certain projects, like [X], where I still don’t fully understand. I just, sort of, don’t know what they’re doing. And… I ought to know what they’re doing.” (A15)*

In depth analysis demonstrated that the abstract description of silos were in essence portrayed similarly to teams. This appeared to be dependent on the context and norms of the person being interviewed; one persons perspective of silos could be classified as another’s close-knit team. Teams were framed positively, as they required the delivery of a project objective, hence team members must work closely together to achieve success. The delivery of CHIP interventions often resulted in close-knit teams, but as the quotes above illustrate, sometimes at the cost of between-group partnerships. From the perspective of other teams, this was then interpreted as the silo-working of others. Therefore, team-working may be considered silo-working when it produces knowledge that is not shared well outside of the group. Those within-team or silo-working practices and experiences are not necessarily made available to other groups within the larger partnership, and this is the main problem we see for some projects in CHIP;

*“They’ve actually gone straight to [the setting] to try and implement something, rather than working through the systems that we’ve already got and the relationships we’ve got with [the setting]. And that has sometimes caused such confusion in [setting] that it’s broken down some relationships.” (P6)*

One participant describes the close knit team-working within their team and yet also discusses being the receiver of negativity from another internal team and how that influenced their perceptions:

*“I think we work really well together. And they’ve got such good knowledge of the people who work in the area…” (A11)*

Which is in stark contrast with:

“…*I’ve had people say to my team…and it wasn’t even that complicated, “so I’ve made this a bit easier for the [A11’s team] people in the room”. And I was like, what?! (Laughs)”. (A11)*

This participant went further to explain that they believed there to be a rift between their teams due to resource issues. One team was long term funded, whereas the other not. This resulted in a perception of different levels of motivation required and demonstrated in the workplace. The interviewee reflected how this had a long-term impact on working with this team “*It’s kind of… scarred us for life”.* This example reflects views from other interviewees in our study, where seemingly minor exchanges can sometimes initiate or expand rifts. In turn this led to increased closeness within teams (pulling themselves closer), and simultaneously pushing other teams away, leading to the perception of silos and silo-working. This example reinforces the idea that working with teams other than your own can be delicate and nuanced work, and that silos can be a matter of perspective.

**Fig. 1:**
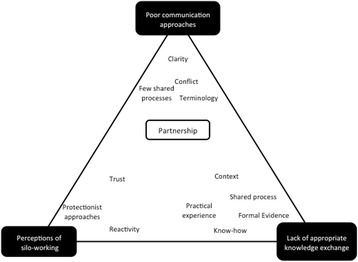
Diagram of independent themes

The figure below presents how three themes fit together conceptually. At the center of each theme is partnership, but you can see that many factors aligned to influence the practice of partnerships in public health (Fig. 1).

## Discussion

We undertook a qualitative study to identify attitudes and experiences of individuals implementing a large and varied public health improvement programme in England. We focused on the identification of the barriers to partnership-working at the managerial level. We found that programme implementation was complicated and context-dependent, its delivery structures were rigid and linear and lacked the capacity for flexible and iterative approaches that were necessary to establish new partnerships or extend older ones. The findings demonstrate that hollow rhetoric could undermine trust between individuals and partnerships. Finally, we found that there were gaps between the values and expectations of different managerial groups and an apparent lack of awareness between members that these gaps existed. These gaps seemed to contribute to the double-edged sword of silo-working. We identified three themes in the data reflecting aspects of communication, appropriate and timely knowledge exchange and the influence of the interpretation of silo-working for partnerships.

### Understanding knowledge exchange in a public health context

Poor communication approaches led to a lack of knowledge exchange that was inappropriate in a range of contexts and situations. This created misunderstandings, eroded trust and acted as a strain on time and scarce resources within the separate organisations. The poor communication approaches we observed reflect the idea of cordial hypocrisy, a term defined as a “strong tendency of people in organisations, because of loyalty or fear, to pretend that there is trust where there is none, being polite in the name of harmony when cynicism and distrust are active poisons, eating away at the very existence of the organization” [10]. In this sense, the individuals in this partnership appeared to work together but were not fully commutated or engaged to the process of establishing good partnerships. There is little surprise that trust is important for public health partnerships [11–15]. While partnership-working that operates with ‘authentic’ trust remains an enigmatic and pivotal challenge, we found evidence that within silos, authentic trust can be found.

We found a lack of appropriate and timely knowledge exchange, rather than a paucity of exchange itself that undermined staff trust and capacity to work together. Expectations surrounding the assumed value of different types of knowledge seemed to influence effective exchange processes, but there was little awareness of these processes and there was little cognizance of approaches to knowledge integration or transfer [16–18]. There appeared to be little knowledge of which type of evidence identified (formal, practical experience and know-how) was most appropriate to use and how and when it would best inform decision-making and day-to-day practices and communications. Higgins et al. [19] work on knowledge exchange in public health similarly reflects the conflict that frontline workers have in prioritising certain kinds of evidence over others, and when each type of evidence can be most valuable. Interventions which advise public health practitioners and managers about the differences, values and capabilities of each type of knowledge described here could improve exchange, particularly those utilising knowledge broker-type roles [2, 18, 20]. Negative perceptions and experience of knowledge exchange resulted in teams turning in on themselves and forming protectionist approaches to their work and relationships in order to meet their own objectives. These insulated approaches resulted in negative perceptions of silo-working across the partnership.

The structure of the CHIP programme compartmentalised work and responsibilities and therefore inadvertently fostered silos. This made projects easier to construct and manage in principle, but harder to implement, and harder to exchange knowledge in practice. Staff struggled to adapt as needed, and share information where required. Axelsson & Axelsson’s description of the fragmentation of responsibilities in inter-organisational working appears similar to the negative aspects of silo-working we discovered. They suggest that work organised in this way leads to efficiency and quality problems of different kinds and this perspective was mirrored in our findings [21, 22].

### Silo-working carries both benefits and risks to partnerships

Silo-working is common in public health practice and is almost universally perceived as a barrier to better working relationships [5, 23–25]. However, we challenge the view that silos are always barriers that must be broken down in order to enable teams and partnerships to function effectively. Our findings demonstrate that categorising silos negatively in this way belies the importance of small, within-team development, and its positive function in the process of partnership working. For example, Ward et al. [2] illustrate naturalistic knowledge exchange activities, proposing that contextualising these activities could focus on natural activities to better engage employees within teams. We have built on this idea by suggesting that some aspects of silo-working are a normal event in the long-term process of team development. We found that silos can be beneficial in a range of ways, for example they can be instrumental (silo-working can allow more efficient action and knowledge exchange) or protectionist (minimising the communication of mistakes or judgement errors, maximising outcomes or outputs achieved) or bureaucratic (maintaining the status quo of established hierarchical power structures).

The range of benefits of silo-working has been demonstrated in other organisational and public health contexts, though not necessarily identified as ‘silo-working’. Riley and Hawe [26] developed a typology of practice narratives in a community based intervention which focused on the social context of practice and staff action within those contexts. Each narrative differed in what was most the most valued endpoint, and the authors note how ‘Implementation of the intervention is not an endpoint in itself’ [26]. Riley’s typology helps to illustrate how, in our study, silo-working can create its own narrative within a group, and in doing so strengthen personal ties and motivate staff to take meaningful action on behalf of themselves, intervention participants, and other staff. However, the challenge remains how best to align the intentions of close-knit groups acting within a silo, and the optimisation of intervention implementation.

A critical insight into why silo-working was a ‘double-edged sword’ in our study comes from Tsoukas [27]. Tsoukas outlines the key elements of organisational social practice as

- being self-referential,
- having a history,
- that members operate and practice in an appreciative system,
- and that it is important to maintain an organisational identity [27].

In our study, the differences between groups at different managerial levels suggest that practices within CHIP groups encompassed all four of the elements of social practice above. Tsoukas’s argument that ‘the management of change in social practices is as much a conceptual as a technical matter’ (p178) is reflected in our findings on silo-working [27]. For example, Tsoukas outlines two elements important for social change that may explain why breaking down silos as barriers was so challenging for CHIP: 1) social practices are language dependent, and 2) if a social system regularly receives information about other systems or its own functioning, it can overcome the tendency of the system to act and resist changes being made (maintaining identity) [27]. In CHIP, these two elements were not clearly defined or communicated between organisations and between organisational groups. Individuals in CHIP struggled to define concepts while implicitly expecting them to be commonly understood, reflecting a weakness expressed in Tsoukas’ first element of change (social practices are language dependent). Second, CHIP staff may have struggled to define these new concepts because of their rapid introduction and a lack of co-operative communication between higher level managers and project and intervention staff, reflecting a weak second element of change (common self-referential practices between the groups).

CHIP practices facilitated and maintained self-referential practice within groups, reflected in the difference between higher level managerial staff concerned with sustaining ideas (instrumental benefits), compared to project manager staff concerned with sustaining their intervention (protectionist benefits) but struggled to develop strong communication systems between organisational and managerial groups (Tsouka’s approach to overcoming barriers to change). This consequently created and sustained gaps in the communication of a “coherent, plausible and legitimate discourse” between groups (p178) [27]. By not being able to overcome the tendency to resist changes (e.g. to terminology, objectives and methods of reporting), silo-working was further entrenched in day to day practice, strengthened teamwork within silos and created a sense of alienation external to a given silo. In this way, Tsoukas’ understanding of how to overcome change in organisational social practices shows the challenge that CHIP struggled to overcome was predominately communication, and we see a lack of informed and dynamic knowledge exchange as a key culprit.

### Public health delivery in the organisational studies context

Literature from organisational studies has examined the challenges of building and working in partnerships across many industrial sectors [28–30]. These studies focus on the practical problems that emerge due to the economic, organisational and individual psychological issues that hamper partnership working. Despite the positive benefits of partnership working and collaboration, evidence suggests that partnerships do not necessarily remove the conflicts that exist in and across project teams [28]. There are often persistent problems in integrating people from different functional areas when lacking formal systems [28]. For example poor communication and knowledge exchange were problems we found in this research, both tasks lacked a formal system across the partnership to improve the situation. There is also limited evidence of the efficacy of formal mechanisms used to develop and promote partnership working, such as financial incentives to collaborate and promotion of teambuilding between groups [28]. According to Dickinson & Glasby [29] the concept of ‘partnership’ brings so much personal and organisational baggage that it often loses its initial appeal and impact within organisations. Their research into health and social care services suggested that partnerships set up without clear desired outcomes and established with underlying motives are always likely to struggle. This was reflected in our study, where discrete silo-working staff conducted work to self-sustain (the protectionist approach), rather than work to form partnerships to achieve and further develop the desired programme outcomes, especially when the gain to them remained unclear. We propose that for our group of public health practitioners, silos were beneficial to their work.

Something that has been touched on previously in the literature is the use of silos as a way of maintaining social identity [30]. In this sense, partnership and collaboration among different groups is obstructed because of the interpersonal relations and operational issues within the smaller silo-entrenched working groups. These issues cover concepts such as group context, power structures, the group norms and values, and the strength of a person’s identification to their silo, which combine to produce different patterns of group behavior which are challenging to overcome [30]. All of these factors were identified in our study, and we found that the staff working within silos, and those perceiving others doing ‘silo-work’ faced difficulties because they failed to take into account the practical, operational and communication issues of the wider partnership, and the positioning of social practices. These are the ‘how to do’s’ of partnership working. Without understanding and planning for the identity of the silos and operationalising the processes and formal mechanisms to ‘conduct’ partnership, it is unlikely that partnership working in complex public health contexts such as this one will be successful, because the silos will continue to be reinforced through the benefits they provide to their members.

Therefore, partnership management that enables teams/silos to maintain their social identity, exchange knowledge and a range of types of evidence in an appropriate and timely manner whilst communicating the wider social, organisational and health contexts of the partnerships they work in may reflect a more realistic, “naturalistic” approach to the process of partnership-working.

### Implications for research and practice

Implications for practice: Knowing when to use which type of knowledge for decision–making, and fostering the acceptance and movement of different types of knowledge across staff grades and teams could enable stronger and more sustainable partnership practices. Communicating the difference between ‘real’ versus ‘ideal’ expectations about what partnership-working or collaborative working can achieve may reduce the risk of further entrenchment of poor partnership-working in public health.

The reframing of silos does not suggest that all aspects of silo-working are positive. Instead it stresses the importance of building on structures of positive interpersonal relationships that exist, rather than stigmatising them as characteristically bad or wholly destructive. Reframing silos, and focusing on the realistic nature of working relationships within teams may enable better partnerships through more accurate perception of what different teams do, and how they do it. This may allow scarce resources and knowledge to be better utilised across groups. The management of perceptions of silo-work is no doubt a challenge, and increasing clarity in communicating terminology, assumptions of vision, objectives and how to achieve them, as well as addressing potential conflicts sooner, rather than later, may help reduce the risk of problematic practices such as cordial hypocrisy from becoming embedded in everyday partnerships in public health.

Implications for research: We support Davies et al. [22] call for more knowledge mobilisation interventions [21]. Current and developing interventions and models in the field of knowledge mobilisation might be of great benefit to public health management in the context of local authority organisations. This is a novel environment that would prove fruitful for investigation of how knowledge sharing across organisational boundaries could foster positive partnerships and overcome the persistent and well identified barriers, including the reframing of silos in public health.

## Conclusions

In this study, poor partnership-working was predicated on three interdependent factors: poor approaches to communication, a lack of understanding of what constitutes appropriate knowledge exchange, and the difficulty of managing perceptions of silo-working and the negative consequences associated with these. Together these three factors undermined the benefits of partnership-working and enhanced the barriers in practice. Reframing silo-working as a normal aspect of the dynamic process of partnership-working may assuage some of the resistance when integrating and using varying sources of knowledge in practice.

Improving the understanding of different and equally useful types of knowledge, may facilitate the mobilisation and integration of different types of knowledge to improve communication and decision-making in public health partnership-working. We contend that silos reflect public health practice that is grounded foremost in social practice, and without recognition and understanding of this, building and sustaining partnerships will remain fraught with misconceptions about silo-work versus team-work and how best to bridge close-knit groups together into stronger partnerships. To reframe silos as necessary and normal part of team development and operation may help to reduce the enduring and negative perception that hinder partnerships and knowledge sharing in public health practice.

## Abbreviations

CHIP: City Health Improvement Programme
OSOP: One Sheet of Paper thematic analysis technique
RE-AIM: Reach, Efficacy/Effectiveness, Adoption, Implementation, Maintenance
